# Cross-Reactivity against *Naja sumatrana* (Black Spitting Cobra) Envenoming from the Haffkine Antivenom in a Mouse Model

**DOI:** 10.1155/2013/247645

**Published:** 2013-08-12

**Authors:** Gregory Cham, Francis Lim, Arul Earnest, Ponnampalam Gopalakrishnakone

**Affiliations:** ^1^Emergency Medicine Department, Alexandra Hospital, 378 Alexandra Road, Singapore 159964; ^2^Singapore Zoological Gardens, 80 Mandai Lake Road, Singapore 729826; ^3^Centre for Quantitative Medicine, Duke-NUS Graduate Medical School, 8 College Road, Singapore 169857; ^4^Venom and Toxin Research Programme, Department of Anatomy, Yong Loo Lin School of Medicine, National University of Singapore, 4 Medical Drive, Block MD10, Singapore 117597

## Abstract

*Naja sumatrana* is the dominant cobra species in Malaysia, Singapore, Borneo, and Sumatra, and it does not have specific antivenom. The Haffkine antivenom has been advocated instead. This study aims to determine the efficacy of this antivenom against *Naja sumatrana* envenoming using a mouse model. *Methods*. Male Swiss albino mice were used. Intravenous LD_50_ was first determined separately for *Naja naja* and *Naja sumatrana* venom. ED_50_ was determined by preincubating antivenom with each venom at 2.5 LD_50_ before administering the mixture into the tail vein. Validation was carried out using a challenge test. Each mouse received 111 *µ*g of *Naja sumatrana* venom intramuscularly followed by intraperitoneal administration of dilute Haffkine antivenom. Survival was recorded 24 hours after envenoming. *Results*. The LD_50_ of *Naja naja* venom was 78.13 *µ*g, standard error (SE) 13.3 *µ*g. The ED_50_ of the Haffkine antivenom against *Naja naja* venom was 45.9 mg, SE 7.5 mg. The LD_50_ and ED_50_ of *Naja sumatrana* venom were 55.5 *µ*g, SE 12.0 *µ*g; and 73.9 mg, SE 12.0 mg, respectively. The intra-peritoneal ED_50_ against 111 *µ*g intramuscular *Naja sumatrana* venom was 136.95 mg, SE 36.74 mg. *Conclusion*. The Haffkine polyvalent antivenom exhibited cross-neutralisation against *Naja sumatrana* venom when used at a higher dose.

## 1. Introduction

### 1.1. Background and Importance

*Naja sumatrana*, commonly known as the black spitting cobra or Equatorial spitting cobra, is the dominant cobra species in Peninsular Malaysia, Singapore, Borneo, and Sumatra [1]. It was estimated that the highest burden of snake bites exists in South Asia, Southeast Asia, and sub-Saharan Africa [2]. Cobra bites are common in Peninsular Malaysia and require significant medical intervention [3, 4]. However, snake bites are considered uncommon in largely urban Singapore [5, 6].

Unfortunately specific antivenom therapy against the *Naja sumatrana* does not exist. The Haffkine antivenom was arbitrarily advocated as an antivenom. It is an equine polyvalent antivenom raised against the Indian binocellate cobra (*Naja naja*), common krait (*Bungarus caeruleus*), Russell's viper (*Vipera russelli*), and saw-scaled viper (*Echis carinatus*) [7]. However, these species of snakes are not normally found in Southeast Asia. The antivenom composition of the Haffkine was chosen for the treatment of black spitting cobra snake bites due to the empirical belief that cobras are originated from the *Naja naja*. These species are, however, considered to be separate now. It is unknown if the Haffkine product has any effective paraspecific activity against the *Naja sumatrana* venom. There have been no previous papers that report the outcomes of patients given this antivenom for black spitting cobra bites.

This study aims to examine if effective cross-neutralisation exists for *Naja sumatrana* envenoming when using the Haffkine antivenom.

## 2. Materials and Methods

### 2.1. Study Design

Using comparable doses of *Naja naja* or *Naja sumatrana* venom, this study analysed the neutralising capability of the Haffkine antivenom in a mouse model. This was followed by a challenge test by giving a fixed dose of *Naja sumatrana* venom intramuscularly and subsequent attempt at rescue with the antivenom.

### 2.2. Materials

*Naja naja* venom was purchased from Accurate Chemical Corporation (New York, USA). *Naja sumatrana* venom was pooled from five local adult *Naja sumatrana* cobras housed at the zoo, lyophilised, and stored at −20°C. The antivenom used was a commercial product from the Haffkine Bio-Pharmaceutical Corporation (Mumbai, India).

### 2.3. Setting and Animal Subjects

Ethics committee approvals from Tan Tock Seng Hospital (TTSH) and Singapore zoo were first sought. The experiments were carried out in the Animal Research Laboratory at TTSH. Only male Swiss albino mice about five weeks of age were used. The mice were bred locally at the Laboratory Animals Centre and weighed between 25.5 and 37.5 grams.

### 2.4. Study Protocol

The animals were quarantined for 24 hours prior to conducting the experiments. Food and water were available ad libitum throughout the study.

#### 2.4.1. Determining the Lethal Dose (LD_50_)

The LD_50_ is the dose of venom required to kill 50% of mice within 24 hours. Each venom was reconstituted by dissolving lyophilised venom in normal saline. Further concentrations were obtained from serial dilutions using additional saline. The lethal toxicity was determined by injecting 0.2 mL of venom, at various concentrations per mouse, into the tail vein [8]. The injections were performed using a 1 mL syringe fitted with a 29-gauge needle. The venom was warmed to 37°C for 30 minutes before injection. Six mice were used for each dilution. The mice were observed for 24 hours.

#### 2.4.2. Determining the Effective Dose (ED_50_)

The ED_50_ is the dose of antivenom required to produce 50% survival of mice within 24 hours when used against intravenous venom at 2.5 LD_50_ for each mouse. The venom and antivenom were incubated prior to intravenous administration. Lyophilised venoms of *Naja naja* and *Naja sumatrana* were obtained from the same batch used for LD_50_ determination. The Haffkine antivenom used to neutralise both types of cobra venom was reconstituted from the same batch. Twofold serial dilution using normal saline was made to obtain additional concentrations of antivenom. Venom at 2.5 LD_50_ and antivenom of various concentrations were mixed and incubated at 37°C for 30 minutes prior to injection. Each mouse was injected with 0.2 mL of venom-antivenom mixture into the tail vein. Four mice were used for each of the dilution groups. The mice were observed for 24 hours.

#### 2.4.3. Haffkine Antivenom versus *Naja sumatrana* Venom Challenge Test

Four groups of four male Swiss albino mice were used. Each was given an intramuscular injection of arbitrarily two times the intravenous LD_50_ into the left thigh muscle from the same batch of reconstituted lyophilised *Naja sumatrana* venom, in 0.05 mL. After two minutes, this was followed by an intraperitoneal administration of Haffkine antivenom at various concentrations, in two mL.

### 2.5. Measurements

The end point for all the above experiments was death of the experimental animal. Total number of deaths was recorded 24 hours after envenoming.

### 2.6. Data Analysis

The LD_50_ and ED_50_ were calculated using the Reed-Muench method [9]. The standard error was calculated according to the method by Pizzi [10]. One-way ANOVA was used to compare the mean weight of the mice for the four groups. Weight was assumed to be normally distributed in the different groups. The Bonferroni method of comparison was used to adjust for the multiple comparisons. Level of significance was set at 5%, and data analysis was carried out using SPSS (Illinois, USA).

## 3. Results

In most of the groups compared the mice were similar in weight. The mean difference in weight for the overall groups of mice was significant (*P* = 0.022, by one-way ANOVA). Pairwise comparisons using the Bonferroni method showed significant difference between the weight of mice used for *Naja naja* ED_50_ and that of *Naja sumatrana* ED_50_ determination (*P* = 0.028). However, the actual difference in the means was only 1.96 g.  Table 1 summarises the weight of the mice used.

The cohort of mice used in the in vivo challenge test was also significantly smaller than those used in the preincubation experiments.

After tail-vein injection of venom, both groups envenomed with *Naja sumatrana* and *Naja naja* venom exhibited weakness and diminished physical activity prior to death.

### 3.1. LD_50_ of Cobra Venom

Survival times diminished with increasing venom doses.  Table 2 summarises the survival of the mice at 24 hours at various *Naja naja* venom concentrations, and Table 3 for the *Naja sumatrana* venom. The LD_50_ of *Naja naja* venom was 78.13 *μ*g, standard error (SE) 13.3 *μ*g. The LD_50_ of *Naja sumatrana* venom was 55.5 *μ*g, SE 12.0 *μ*g.

### 3.2. ED_50_ of the Haffkine Antivenom against Cobra Venom

Results of the preincubation experiments are summarised in Tables 4 and 5, for *Naja naja* and *Naja sumatrana*, respectively. The data showed a similar dose-response relationship, with improved survival on higher doses of antivenom, with the venom load fixed at 2.5 LD_50_. The ED_50_ of the Haffkine antivenom against *Naja naja* venom was 45.9 mg, SE 7.5 mg. The ED_50_ of the Haffkine antivenom against *Naja sumatrana* venom was 73.9 mg, SE 12.0 mg.

### 3.3. The ED_50_ of Intraperitoneal Haffkine Antivenom versus Intramuscular *Naja sumatrana* Venom

After the initial intramuscular envenoming, the mice exhibited paralysis in the left leg but continued to be mobile. The paralysis was recovered gradually particularly among the survivors.  Table 6 summarises the survival of the mice at different concentrations of antivenom. The intraperitoneal ED_50_ against 111 *μ*g intramuscular *Naja sumatrana* envenoming was 136.95 mg, SE 36.74 mg.

There was no local tissue destruction over the site of injection 24 hours later among the surviving mice.

## 4. Discussion

There have been no published studies addressing antivenom therapy in *Naja sumatrana* envenoming, but this species represents the dominant venomous snake in this region. Beyond this region, the species might also be found in zoos or in private collections.

The Haffkine polyvalent antivenom exhibits effective cross-neutralisation against *Naja sumatrana* venom, revealing a stepwise improvement in survival with increasing antivenom dose. The ED_50_ was higher than the usual effective dose required for *Naja naja* neutralisation in preincubation experiments. However, it is not clear which fraction or a combination of fractions of the *Naja naja*, *Bungarus caeruleus*, *Vipera russelli*, or *Echis carinatus* antibodies that drove the results we obtained. An in vivo challenge using intraperitoneal Haffkine antivenom, given following the intramuscular *Naja sumatrana* envenoming, also showed a stepwise dose response as the dose of antivenom was increased.

The quality of snake venom varies between individual snakes and within the same snake milked on different occasions. To improve the consistency of the venom and minimise stress to the snakes, the Cobras were milked in a single sitting, mixed, and lyophilised. Likewise, the antivenoms used were checked to ensure they belong to the same batch in manufacture.

LD_50_ experiments sought to standardise the lethal dose for each venom in this particular mouse model. ED_50_ experiments compared the neutralising capacity of a standard antivenom against a comparable venom load. These parameters were useful in the present cross-neutralisation study, and would also prove useful in future antivenom or intervention studies on the subject.

Preincubation experiments were chosen initially to investigate if the Haffkine antivenom had any efficacy against the *Naja sumatrana* venom. This was considered better than gel diffusion experiments as apparent reactions in gel diffusion plates occur in vitro and may not be uniformly associated with survival. It was anticipated that there would be immediate bioavailability if antivenom was given intravenously; but if venom was administered intramuscularly or subcutaneously, it would have slower onset, more sustained effect, and could remain lethal after the effects of intravenous antivenom have worn off. Preincubation experiments were chosen as this technique would control the differences in venom and antivenom bioavailability that may confound the results. Only male mice were used to avoid intergender difference in survival if any.

To validate the results, an in vivo challenge test was considered necessary to simulate a clinical situation. The intraperitoneal ED_50_ of the Haffkine antivenom against *Naja sumatrana* was indeed higher than the ED_50_ in preincubation studies due to the reduced bioavailability of intraperitoneal antivenom compared to the inherently efficient intravenous availability of antivenom in preincubation studies. In anticipation of this, the higher dosages of antivenom required would not be easily miscible in the small volumes compatible with intravenous tail-vein administration. The intraperitoneal route was chosen, as it allowed for greater volumes for antivenom dilution to occur. An intramuscular route of envenoming more closely resembles what happens clinically. The intramuscular envenoming followed by a single dose of intraperitoneal antivenom exhibited compatible pharmacokinetic properties to allow for the observations made in the in vivo challenge tests.

The LD_50_ varies according to the route of administration, and the intramuscular LD_50_ was not determined. In the in vivo challenge test, twice the preincubation *Naja sumatrana* intravenous LD_50_ dose (111 *μ*g) was arbitrarily used intramuscularly, while we explored the ED_50_. The choice of this dose did not affect the comparisons which were made between groups of mice that differed only in the intraperitoneal doses of the antivenom. 

The mice weights were largely comparable between different cohorts. The 1.96 g difference in mean weights between the mice used for *Naja naja* ED_50_ and *Naja sumatrana* ED_50_ determination was not significant in practice. It was more crucial for the mice used for LD_50_ and its corresponding ED_50_ for the same venom to be comparable.

The venom of the *Naja sumatrana* shares similar composition with *Naja naja* [11, 12]. Cobras have L-amino acid oxidase, phospholipase A2, protease, phosphodiesterase, 5′-nucleotidase, alkaline phosphomonoesterase, hyaluronidase, and acetylcholinesterase in common. These similarities in the lethal components of the venoms may explain the paraspecific activity of the Haffkine antivenom against *Naja sumatrana* venom. However, regional differences in venom composition may limit the explanation that could be derived from the similarities in venom composition [13].

## 5. Limitations and Future Questions

This experiment was conducted using mice, and the difference between their metabolism and that of humans is significant, but the results would probably hold true for humans. The findings should be correlated with clinical observations in humans. A higher dose of 1.6 times was determined using pre-incubation experiments, and it would likely not apply in a clinical situation.

The observations were made over 24 hours, focusing on the acute lethal effects of *Naja sumatrana* venom from neuromuscular paralysis. Beyond 24 hours, the venom could remain lethal from other mechanisms, for example, haematological disorders not studied in this experiment.

The experiment reveals effective paraspecific activity against paralysis, between Haffkine antivenom in *Naja sumatrana* envenoming, when used at a higher dose in this model. The Haffkine is a viable choice of antivenom available to address *Naja sumatrana* envenoming until specific antivenom is developed, and treatment could be instituted at more efficient and perhaps safer doses.

## Figures and Tables

**Table 1:** Weights of the mice compared between groups.

	*n*	Mean weight (grams)	Std. deviation	Range
Group for *Naja naja* LD_50_ determination	24	29.98	0.99	27.5–32.0
Group for *Naja sumatrana* LD_50_ determination	24	29.42	2.41	25.5–37.5
Group for *Naja naja* ED_50_ determination	12	28.50	0.95	27.0–30.5
Group for *Naja sumatrana* ED_50_ determination	12	30.46	1.14	28.0–32.0

Total	72	29.62	1.72	25.5–37.5

**Table 2:** 24-hour-survival of mice after tail-vein envenoming with *Naja naja* venom.

Venom (*μ*g)	Lived	Died	Mortality %
19.5	6	0	0.00
39.1	6	0	0.00
78.1	3	3	50.00
156.3	0	6	100.00

**Table 3:** 24-hour-survival of mice after tail-vein envenoming with *Naja sumatrana* venom.

Venom (*μ*g)	Lived	Died	Mortality %
19.5	6	0	0.00
39.1	4	2	28.57
78.1	1	5	87.50
156.25	0	6	100.00

**Table 4:** 24-hour-survival of mice after tail-vein injection of Haffkine antivenom incubated with 2.5 LD_50_ *Naja naja* venom.

Antivenom dose (mg)	Lived	Died	Mortality %
65.0	4	0	0.00
32.5	0	4	100.00
16.2	0	4	100.00

**Table 5:** 24-hour-survival of mice after tail-vein injection of Haffkine antivenom incubated with 2.5 LD_50_ *Naja sumatrana* venom.

Antivenom dose (mg)	Lived	Died	Mortality %
104.6	4	0	0.00
52.3	0	4	100.00
26.1	0	4	100.00

**Table 6:** 24-hour-survival of mice after intra-peritoneal Haffkine antivenom following 111 *μ*g intramuscular *Naja sumatrana* envenoming.

Antivenom Dose (mg)	Lived	Died	Mortality %
192.14	3	1	14.29
147.8	2	2	50.00
103.46	1	3	85.71
59.12	0	4	100.00
